# Supplementary material for: Genomic signatures of migratory preference and historical whaling in eastern South Pacific humpback whales
Source: Commun Biol. 2026 Apr 15;9:630. doi: 10.1038/s42003-026-10037-x (PMC13161203; doi:10.1038/s42003-026-10037-x)
Supplement: Supplementary file 2 — Supplemental Information for: Genomic signatures of migratory preference and historical whaling in eastern South Pacific humpback whales [file 42003_2026_10037_MOESM2_ESM.pdf]

Supplemental Information for:

Genomic signatures of migratory preference and historical whaling in eastern South Pacific humpback whales

Enrique Celemín, Jorge Acevedo, Linda Hagberg , Cristina Castro, Juliana Castrillón, Pedro Valenzuela, Luis A. Pastene, Ralph Tiedemann

Table of Contents:

**Supplementary Figure 1:** Bioinformatic pipeline	Page 2
**Supplementary Figure 2:** NGSAdmix analysis K1-5	Page 3
**Supplementary Figure 3:** ΔK (Evanno method)	Page 4
**Supplementary Figure 4:** Mitochondrial phylogenetic tree	Page 4
**Supplementary Figure 5:** Nuclear – mitochondrial genome clustering comparison	Page 5
**Supplementary Figure 6:** Fit of the SFS of the *δaδi* models to the observed SFS from the data	Page 5
**Supplementary Figure 7:** SMC++ analysis for the different regions	Page 6
**Supplementary Figure 8:** GONE analysis for the different regions	Page 7
**Supplementary Figure 9:** Single-population demographic models tested with *δaδi*	Page 8
**Supplementary Table 1:** Sample information	Page 9
**Supplementary Table 2:** Fst values among grounds	Page 11
**Supplementary Table 3:** Summary of the five demographic models' performances	Page 11
**Supplementary Table 4:** Likelihood ratio test (LRT) between nested single-population models	Page 11
**Supplementary Table 5:** Demographic parameter estimates for the 2-epoch and 3-epoch models.	Page 12
**Supplementary Table 6:** Runs of homozygosity statistics for each sample	Page 13

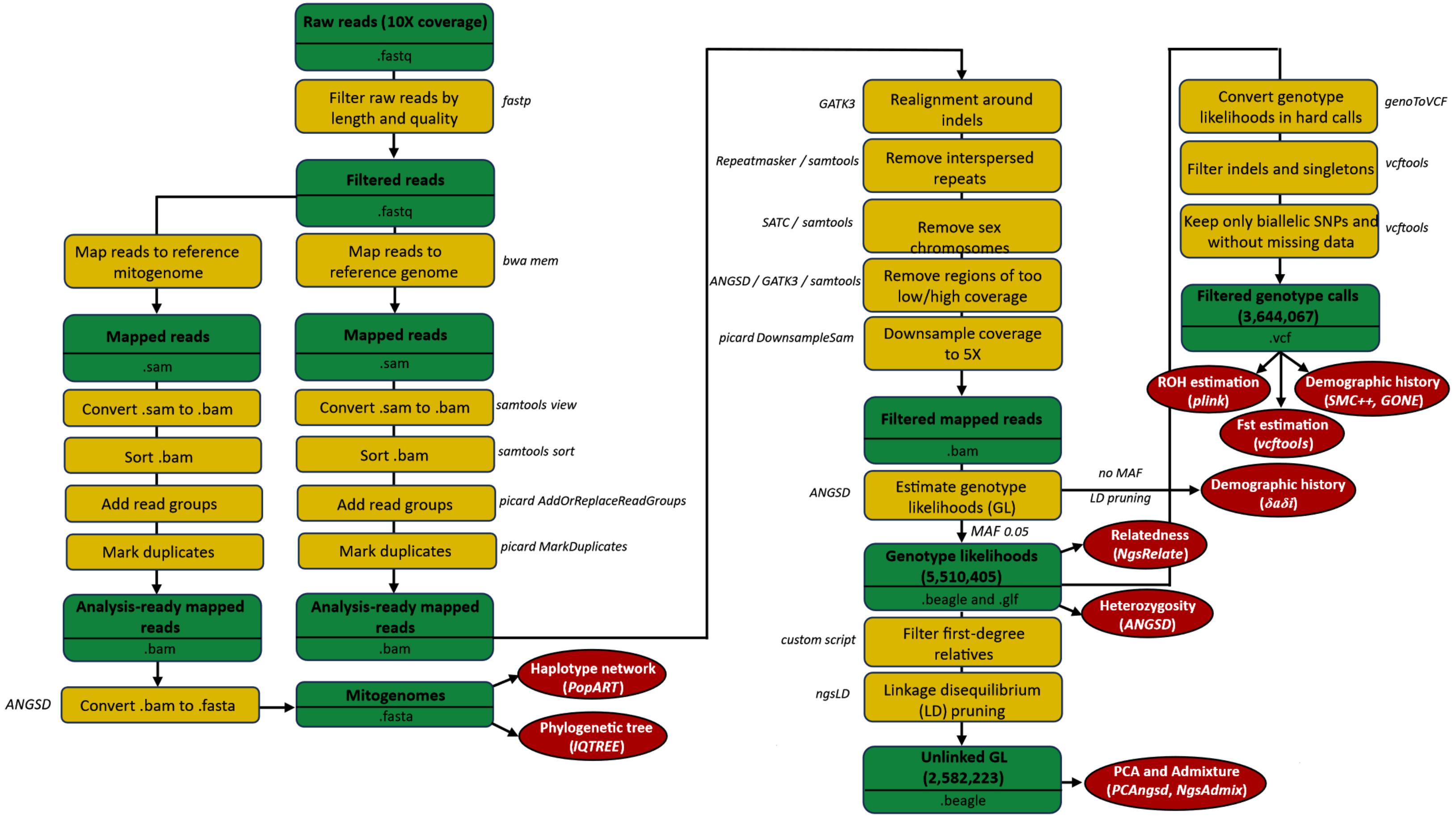

Supplementary Figure 1: Bioinformatic pipeline. Green boxes represent raw, intermediate and final files; yellow boxes show the different processing steps and next to them the software used; and red circles represent the different analysis performed

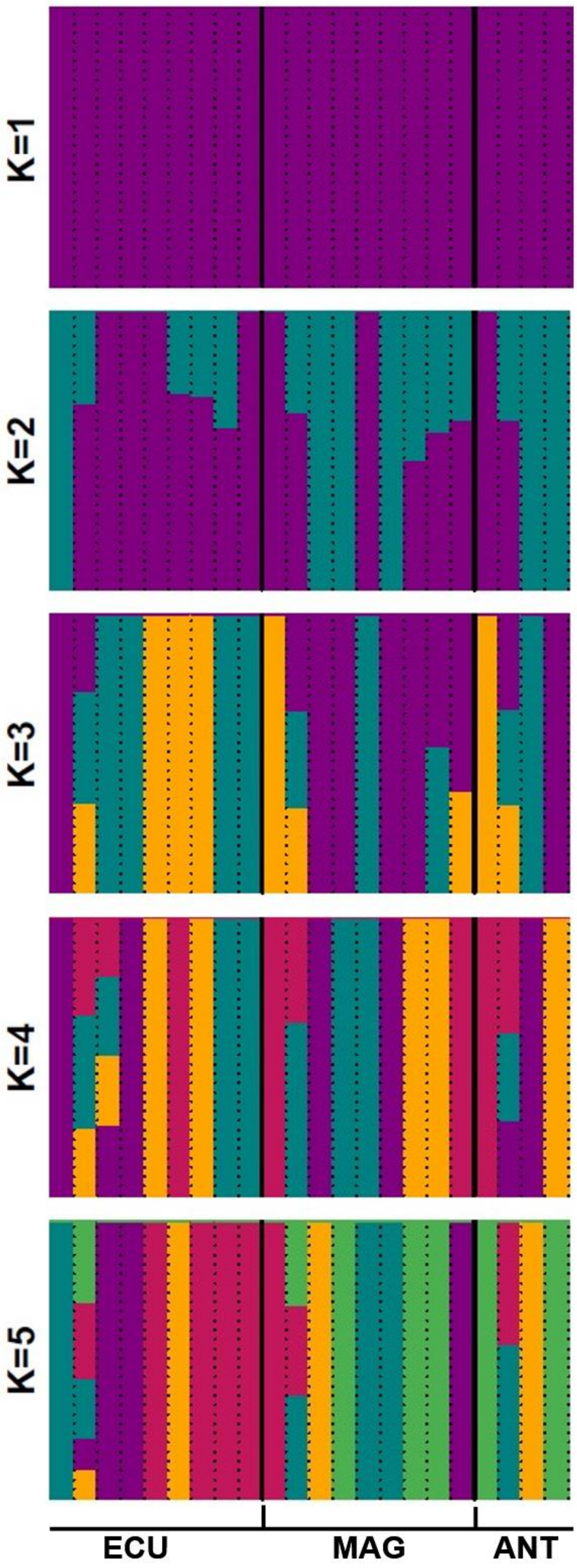

Supplementary Figure 2: Admixture analysis of the dataset without first and second-degree relatives (N = 22) showing K=1-5. Each small vertical bar represents a specimen and the colouring corresponds to its nuclear genetic ancestry

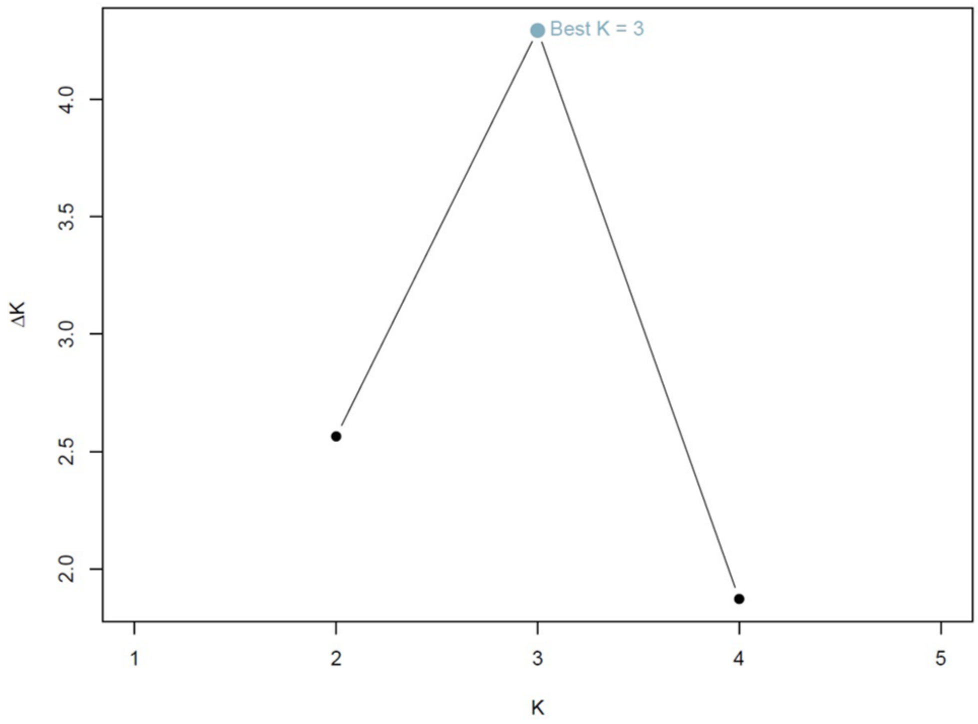

Supplementary Figure 3: ΔK (Evanno method) plotted against K to infer the most likely number of clusters. Note that ΔK could not be calculated for for K = 1 and K = 5.

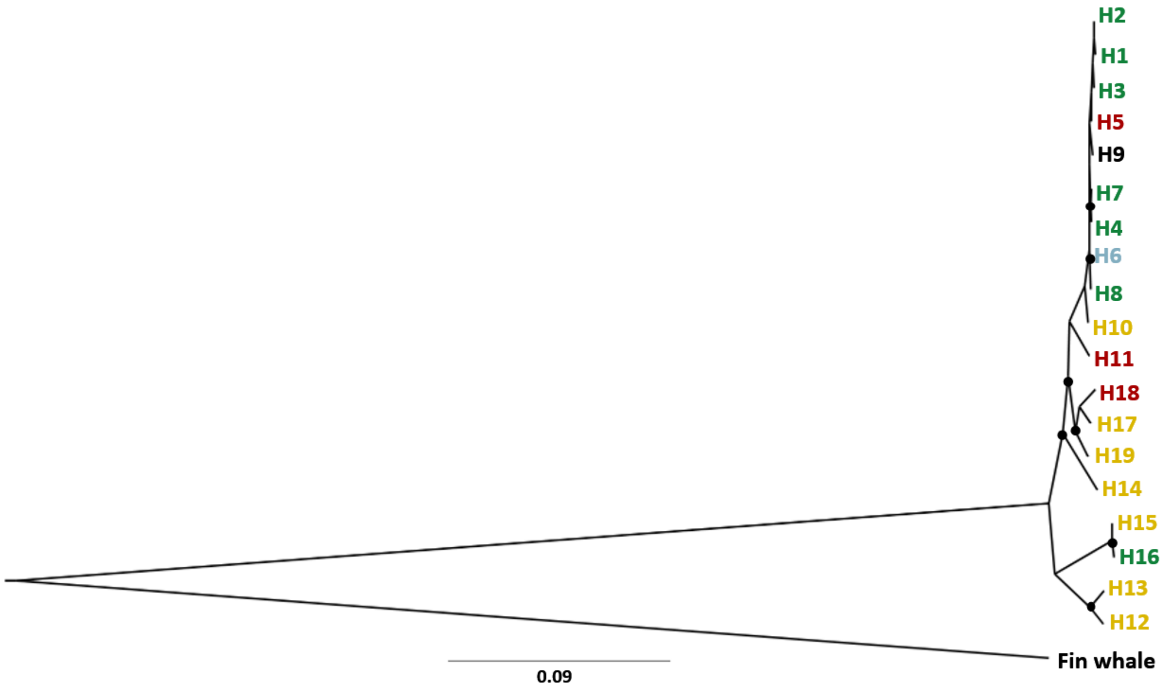

Supplementary Figure 4: Mitochondrial genome phylogenetic tree of the different identified haplotypes. The scale bar represents 0.009 substitutions per site. Black circles show bootstrap values > 90. The colouring corresponds to different sampling locations: Ecuador breeding ground in yellow, Magellan strait feeding ground in green and Antarctic Peninsula feeding ground in red. Please note that H6 as was found in the three grounds was coloured differently in light blue.

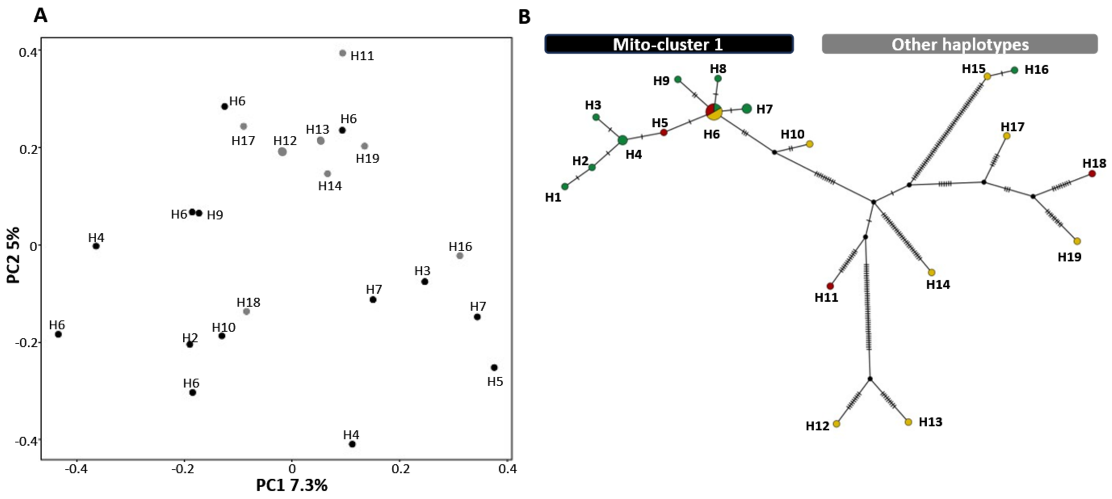

Supplementary Figure 5: Nuclear – mitochondrial genome clustering comparison. (A) Principal component analysis (PCA) of the dataset without second-degree relatives ($N = 22$) showing the mitogenome haplotype of every individual. The colouring corresponds to the mitogenome haplotype network, the monophyletic Mito-cluster 1 in black and the remaining haplotypes in grey. (B) Mitochondrial haplotype median-joining network for Humpback whales. Ticks along the branch lengths denote nucleotide differences and colours indicate sampling location: Ecuador breeding ground in yellow, Magellan Strait feeding ground in green and Antarctic Peninsula feeding ground in red. Note that H1, H8, and H15 are not shown in the PCA because they were detected in a related individual that was filtered out during the nuclear population structure analysis.

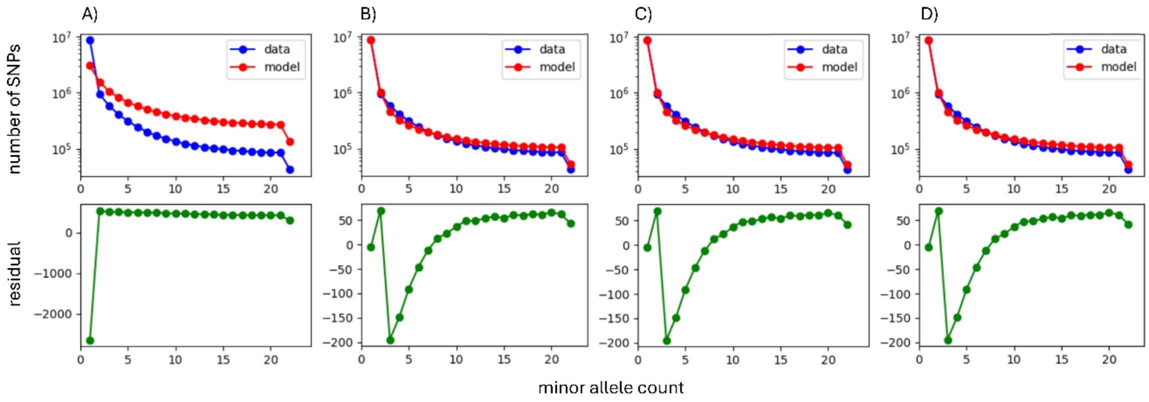

Supplementary Figure 6: Diagnostic plots for the four converging ∂a∂i models. A) 1-epoch model; B) 2-epoch model; C) 3-epoch model; D) 3-epoch model with most recent size change (T_2) fixed to 5 generations ago. The upper panel of each subplot shows the site frequency spectrum (SFS) of the genomic data (blue) and the inferred two-population SFS from the maximum-likelihood iteration of the given model (red); in the lower panel we show the residuals between model and data as calculated in ∂a∂i. Note the high similarity of model fits in B), C), and D). Inferences are based on n=22.

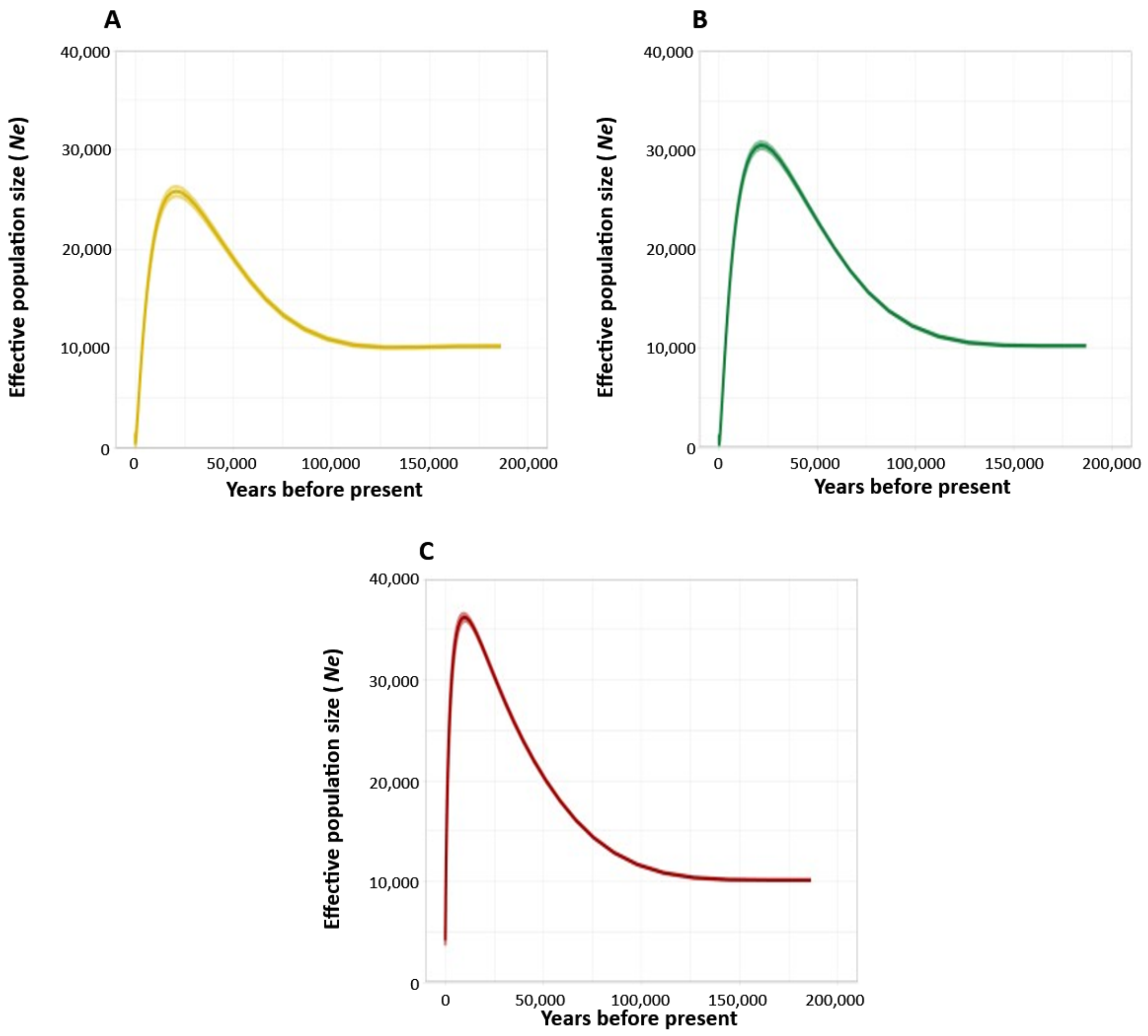

Supplementary Figure 7: Changes in historical effective population size (Ne) through time inferred with the coalescence method applied in SMC++ for (A) Ecuador breeding ground (n=9), (B) Magellan Strait feeding ground (n=9), and (C) Antarctic Peninsula feeding ground (n=4). Solid Ne curves depict the geometric mean over 100 independent estimates, while blurred lines represent the 95% confidence intervals. The X-axis represents time before present in years while the Y-axis depicts Ne.

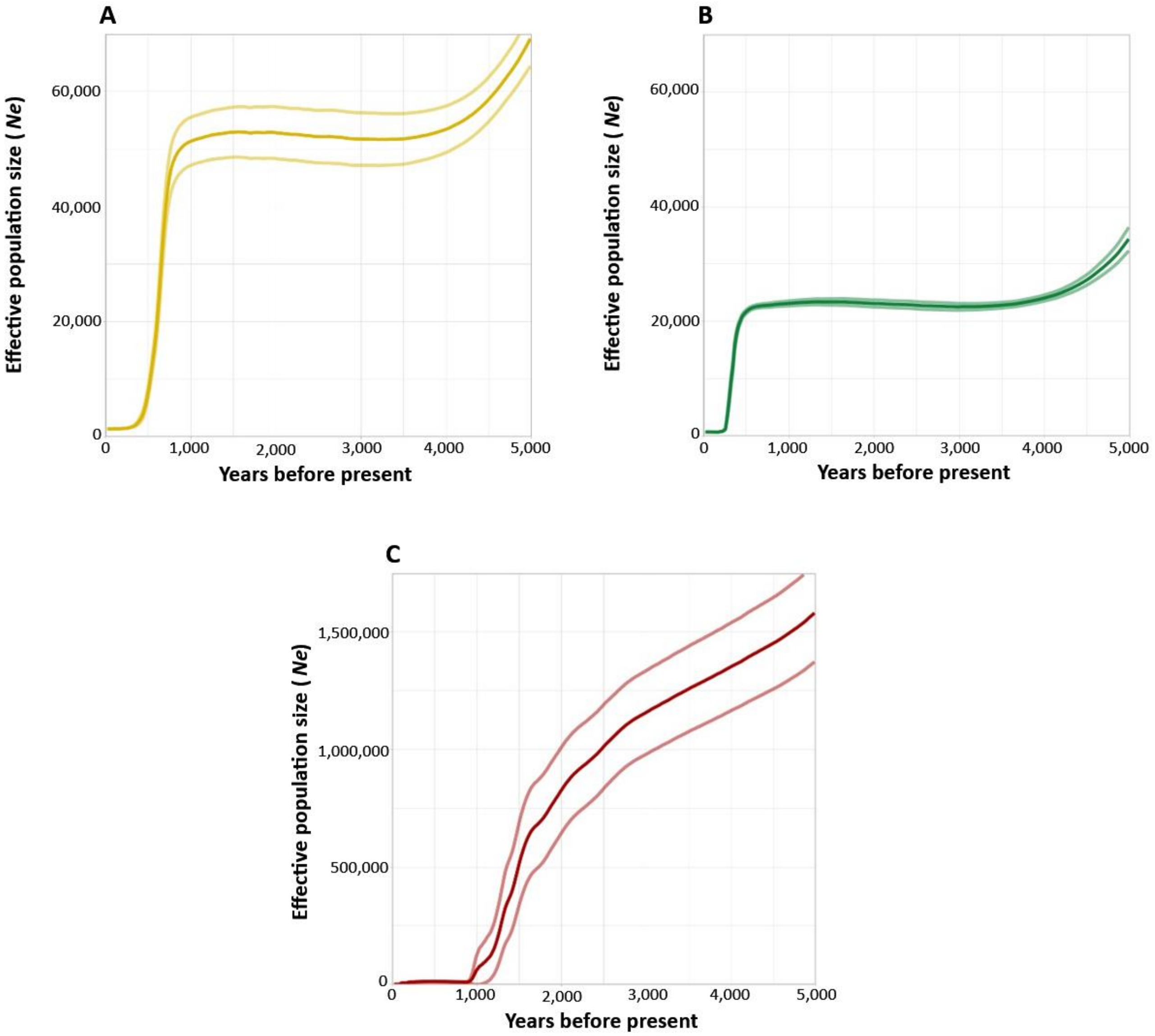

Supplementary Figure 8: Changes on recent effective population size (Ne) through time inferred with the linkage disequilibrium approach implemented in GONE for (A) Ecuador breeding ground (n=9), (B) Magellan Strait feeding ground (n=9), and (C) Antarctic Peninsula feeding ground (n=4). Solid Ne curves depict the geometric mean over 100 independent estimates, while blurred lines represent the 95% confidence intervals. The X-axis represents time before present in years while the Y-axis depicts Ne.

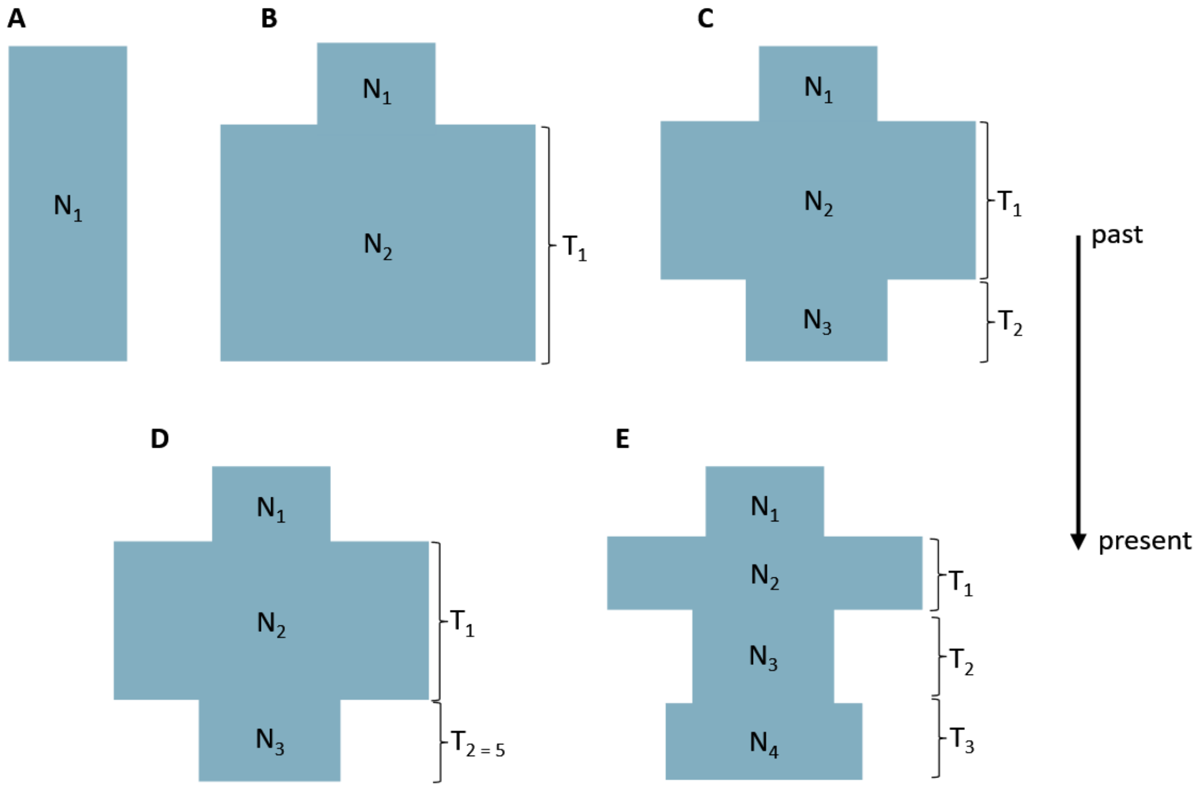

Supplementary Figure 9: Single-population demographic models tested with *δaδi*. All models are forward in time. For population size parameters (N_1,N_2,N_3, and N_4), values represent numbers of diploids. For time parameters (T_1, T_2 and T_3), values represent the number of generations. The sizes or duration of parameters are not to scale, but the directions of population size changes are presented according to the inferred demographic scenarios (except for the 4-epoch model that were not calculated). Note that our models only forces the occurrence of size change among epochs, not its direction. (A) 1-epoch model with no population size change. (B) 2-epoch model with one size change event, from the ancestral size (N_1) to the current size (N_2) occurring T_1 generations ago. (C) 3-epoch model with two size change events, from the ancestral size (N_1) to an intermediated size (N_2) occurring T_1 generations ago to the current size (N_2) occurring T_2 generations ago. (D) same 3-epoch model as before but fixing T_2 to five generations, which corresponds roughly to when most of the whaling activity began (~1,920). As sensitivity test, this model was re-run with other values for T_2, i.e. 1, 10, 20, and 30. (E) 4-epoch model with three size change events, from the ancestral size (N_1) to an intermediate size (N_1) occurring T_1 generations ago to another intermediate size (N_2) occurring T_2 generations ago to the current size (N_3) occurring T_3 generations ago.

Supplementary Table 1: Sample information indicating the sample ID (* indicates the samples that were removed from the dataset without first degree relatives and # the samples that were removed from the dataset without second-degree relatives), sex, sampling region, coordinates, sampling date, number of raw sequencing reads, percentage of reads kept after filtering, percentage of mapped reads, final coverage before downsampling and SNPs called per individual. ANT = Antarctic Peninsula; ECU = Ecuador; MAG = Magellan Strait.

Sample ID	Sex	Region	Latitude	Longitude	Date	#Raw reads	%Reads kept	%Reads mapped	Final coverage	SNPs called
**MnAnt02**	F	ANT	-64°49´06	-62°47´17	26/02/2010	202995606	99.11	99.87	5.86	5328087
**MnAnt03**	F	ANT	-65°13´58	-64°08´27	02/03/2010	194728952	98.94	99.87	5.69	5352912
**MnAnt04 *#**	F	ANT	-65°13´58	-64°08´27	02/03/2010	161142408	99.15	99.83	5	5286030
**MnAnt07**	F	ANT	-65°20´47	-64°10´34	02/03/2010	208041204	98.95	99.90	6.14	5349497
**MnAnt12**	F	ANT	-65°26´39	-64°02´08	05/03/2010	200112398	99.00	99.86	5.79	5344518
**MnEcu01**	F	ECU	-1.25	-81.06	24/09/2022	178554902	98.88	99.87	5.11	5334429
**MnEcu06**	F	ECU	-1.23	-80.84	29/09/2022	174086088	98.79	99.72	5.09	5307899
**MnEcu09 #**	M	ECU	-1.24	-80.83	29/09/2022	191539170	98.94	99.76	6.06	5349619
**MnEcu14**	F	ECU	-1.41	-80.77	30/09/2022	164498964	99.02	99.88	5	5303613
**MnEcu19**	F	ECU	-1.3	-81.05	11/02/2023	223897818	99.00	99.86	6.84	5356684
**MnEcu30**	M	ECU	-1.31	-81.02	29/08/2023	326481878	98.80	99.93	9.17	5348671
**MnEcu32**	M	ECU	-1.58	-80.87	30/08/2023	297129298	98.68	99.92	8.79	5343927
**MnEcu33**	M	ECU	-1.58	-80.88	30/08/2023	329444594	98.70	99.92	9.37	5353019
**MnEcu37**	M	ECU	-1.3	-81.06	01/09/2023	279252044	99.07	99.95	9.5	5311534
**MnEcu39**	M	ECU	-1.31	-81.06	01/09/2023	273172072	98.92	99.94	8.59	5346674
**MnMg136**	F	MAG	53.33	72.18	11/02/2022	276394492	98.83	99.67	8.42	5336892
**MnMg137**	M	MAG	53°50.633	72°11.818	12/02/2022	179594682	99.02	99.83	6.07	5343036
**MnMg139 #**	M	MAG	-53.8386	-72.2011	23/04/2022	183465370	98.98	99.79	6.04	5347432
**MnMg143**	F	MAG	-53.85	-72.18	22/02/2023	179284100	99.07	99.87	5.9	5339679
**MnMg146**	M	MAG	53°51.228	72°11.868	23/03/2023	200563772	99.03	99.77	6.41	5347946
**MnMg147**	M	MAG	-53.85	-72.22	23/03/2023	204436950	98.96	99.80	6.57	5348027

**MnMg149**	F	MAG	53°50.927	72°11.958	24/03/2023	201039744	98.94	99.87	6.4	5353232
**MnMg151**	M	MAG	53°39.626'	72°14.348'	26/03/2023	178033464	99.07	99.88	5.92	5330734
**MnMg152**	F	MAG	53°39.616'	72°14.523'	26/03/2023	206267134	99.05	99.73	6.44	5340928
**MnMg153 *#**	F	MAG	53°39.547'	72°14.468'	26/03/2023	167153506	99.09	99.82	5.45	5322763
**MnMg154**	M	MAG	53°39.492'	72°14.486'	26/03/2023	173542164	99.03	99.87	5.88	5328653

Supplementary Table 2: Pairwise genetic differentiation (weighted Weir and Cockerham Fst) among humpback whale populations from Ecuador (ECU), Magellan Strait (MAG) and Antarctica (ANT). Negative values were interpreted as zero.

	ECU	MAG	ANT
**ECU**	-	-	-
**ANT**	0.00005	-	-
**MAG**	0.00196	-0.00138	-

Supplementary Table 3: Summary of the demographic models' performances. K: number of estimated parameters. Log-likelihood: best log-likelihood obtained for each model, measuring the fit of the model to the empirical data. AIC: Akaike information criterion is calculated using the equation: *AIC = 2*K - 2*Log-likelihood*. Convergence of parameters and likelihoods is reported in the last column.

Model	K	Log-likelihood	AIC	Converged
**1-epoch**	0	-5639653.08	11279308.16	Yes
**2-epoch**	2	-58085.15	116174.3	Yes
**3-epoch**	4	-58091.35	116190.7	Yes
**3-epoch_ T_{BOT}=1**	3	-58106.82	116219.64	Yes
**3-epoch_T_{BOT}=5**	3	-58153.49	116312.98	Yes
**3-epoch_ T_{BOT}=10**	3	-58176.56	116359.12	Yes
**3-epoch_ T_{BOT}=20**	3	-58119.71	116245.42	Yes
**3-epoch_ T_{BOT}=30**	3	-58137.37	116280.74	Yes
**4 epoch**	6	-544.44	1100.88	No

Supplementary Table 4: Likelihood ratio test (LRT) between nested models, calculated using the formula: *-2 * [log likelihood (simple) – log likelihood (complex)]*. LRT significance was evaluated using a chi-square test without multiple comparison adjustment with two degrees of freedom (number of parameter differences between models). The 2-epoch model is significantly better than the 1-epoch model, and the 4-epoch model significantly better than the 3-epoch model, while a 3-epoch model is not better than the 2-epoch model. (P-values from X^2 test: $p_{2vs1epochs} = 0$, $p_{3vs2epochs} = 1$, $p_{4vs3epochs} = 0$).

	1-epoch	2-epoch	3-epoch
**1-epoch**	-	-	-
**2-epoch**	11167305 ***	-	-
**3-epoch**	-	-0.32639	-

Supplementary Table 5: Demographic parameter estimates for the most likely models, (2-epoch and 3- epoch). Population size parameter (N) values are in units of number of diploids. Time parameter (T), values are in units of generations. * denotes the parameter was fixed.

Model	Parameter	Estimate	95% CI
**2-epoch**	N_1	3,692.85	3,688.36 – 3,697.33
	N_2	394,292.32	393,909.12 – 394,675.52
	T_1	2,485.29	2,482.15 – 2,488.43
**3-epoch**	N_1	3,692.15	3,687.83 – 3,696.47
	N_2	393,191.94	392,763.76 – 393,620.11
	N_3	2,444.57	2,103.15 – 2,785.27
	T_1	2,486.29	2,482.12 – 2,489.73
	T_2	0	NA
**3-epoch_$_{TBOT}$**=1	N_1	3,694.75	3,688.25 – 3,701.25
	N_2	395,131.51	394,732.97 – 395,530.04
	N_3	12,360.79	11,203.44 – 13,518.14
	T_1	2,484.45	2,480.87 – 2,488.02
	T_2*	1	NA
**3-epoch_$_{TBOT}$**=5	N_1	3,690.72	3,685.45 – 3,695.98
	N_2	397,868.80	397,209.13 – 398,528.46
	N_3	19,418.35	17,389.51 – 21,447.19
	T_1	2,485.83	2,482.56 – 2,489.10
	T_2*	5	NA
**3-epoch_$_{TBOT}$**=10	N_1	3,698.16	3,686.14 – 3,710.19
	N_2	388,460.77	387,168.50 – 389,753.03
	N_3	42,223.06	38,878.75 – 45,567.36
	T_1	2,486.91	2,483.25 – 2,490.56
	T_2*	10	NA
**3-epoch_$_{TBOT}$**=20	N_1	3,691.65	3,687.24 – 3,696.05
	N_2	398,600.69	397,591.00 – 399,610.37
	N_3	128,046.96	114,067.60 – 142,026.32
	T_1	2,484.26	2,480.37 – 2,488.1
	T_2*	20	NA
**3-epoch_$_{TBOT}$**=30	N_1	3,690.33	3,684.95 – 3,695.71
	N_2	398,403.23	397,424.41 – 399,382.05
	N_3	121,361.04	108,035.65–134,686.44
	T_1	2,485.11	2,481.88 – 2,488.35
	T_2*	30	NA

Supplementary Table 6: Runs of homozygosity statistics for each sample indicating the sampling region, total length of the genome under ROHs, length of the genome under short ROHs (0.1-0.3 Mb), length of the genome under medium ROHs (0.3-1 Mb), ROH length average (Kb), length of the genome under long ROHs (>1 Mb) and total fraction of the genome under ROHs (FROH),

Sample	Region	Mb ROH	Mb short ROH	Mb medium ROH	Mb long ROH	Kb ROH length mean	FROH (all)
**MnAnt02**	ANT	193.92	156.30	34.80	2.81	173.60	0.132
**MnAnt03**	ANT	153.72	119.42	29.18	5.12	174.29	0.104
**MnAnt04**	ANT	147.21	120.45	26.76	0.00	169.40	0.100
**MnAnt07**	ANT	190.43	144.56	43.09	2.78	177.80	0.129
**MnAnt12**	ANT	115.15	102.65	12.50	0.00	159.49	0.078
**MnEcu01**	ECU	159.35	124.31	30.31	4.73	174.53	0.108
**MnEcu06**	ECU	138.64	118.61	17.47	2.56	162.91	0.094
**MnEcu09**	ECU	164.21	129.99	31.58	2.64	176.01	0.111
**MnEcu14**	ECU	148.14	124.41	18.80	4.93	165.15	0.100
**MnEcu19**	ECU	142.39	118.27	21.96	2.15	163.48	0.097
**MnEcu30**	ECU	182.27	153.15	29.12	0.00	165.70	0.124
**MnEcu32**	ECU	196.94	146.00	40.38	10.56	181.68	0.134
**MnEcu33**	ECU	198.86	153.38	40.99	4.49	178.19	0.135
**MnEcu37**	ECU	207.63	142.63	53.08	11.92	193.15	0.141
**MnEcu39**	ECU	241.75	168.83	53.81	19.11	192.78	0.164
**MnMg136**	MAG	162.60	131.38	28.38	2.85	170.62	0.110
**MnMg137**	MAG	145.41	106.88	33.89	4.64	180.18	0.099
**MnMg139**	MAG	147.57	120.90	23.17	3.49	170.01	0.100
**MnMg143**	MAG	148.45	119.67	24.88	3.90	173.83	0.101
**MnMg146**	MAG	161.76	131.11	25.33	5.32	170.27	0.110
**MnMg147**	MAG	168.95	137.75	27.81	3.39	171.00	0.115
**MnMg149**	MAG	189.10	137.66	49.42	2.02	182.35	0.128
**MnMg151**	MAG	167.74	139.51	24.23	4.00	168.41	0.114
**MnMg152**	MAG	102.12	88.40	12.71	1.00	157.35	0.069
**MnMg153**	MAG	96.37	84.08	12.29	0.00	158.25	0.065
**MnMg154**	MAG	153.10	124.76	28.34	0.00	171.83	0.104
