# Supplementary material for: Genomic signatures of migratory preference and historical whaling in eastern South Pacific humpback whales
Source: Commun Biol. 2026 Apr 15;9:630. doi: 10.1038/s42003-026-10037-x (PMC13161203; doi:10.1038/s42003-026-10037-x)
Supplement: Supplementary file 3 — Reporting Summary [file 42003_2026_10037_MOESM3_ESM.pdf]

nature portfolio

Corresponding author(s): Ralph Tiedemann

Last updated by author(s): 27/3/2026

Reporting Summary

Nature Portfolio wishes to improve the reproducibility of the work that we publish. This form provides structure for consistency and transparency in reporting. For further information on Nature Portfolio policies, see our Editorial Policies and the Editorial Policy Checklist.

Statistics

For all statistical analyses, confirm that the following items are present in the figure legend, table legend, main text, or Methods section.

n/a	Confirmed	
☐	☒	The exact sample size (*n*) for each experimental group/condition, given as a discrete number and unit of measurement
☐	☒	A statement on whether measurements were taken from distinct samples or whether the same sample was measured repeatedly
☐	☒	The statistical test(s) used AND whether they are one- or two-sided<br>*Only common tests should be described solely by name; describe more complex techniques in the Methods section.*
☒	☐	A description of all covariates tested
☐	☒	A description of any assumptions or corrections, such as tests of normality and adjustment for multiple comparisons
☐	☒	A full description of the statistical parameters including central tendency (e.g. means) or other basic estimates (e.g. regression coefficient) AND variation (e.g. standard deviation) or associated estimates of uncertainty (e.g. confidence intervals)
☐	☒	For null hypothesis testing, the test statistic (e.g. *F*, *t*, *r*) with confidence intervals, effect sizes, degrees of freedom and *P* value noted<br>*Give P values as exact values whenever suitable.*
☐	☒	For Bayesian analysis, information on the choice of priors and Markov chain Monte Carlo settings
☒	☐	For hierarchical and complex designs, identification of the appropriate level for tests and full reporting of outcomes
☒	☐	Estimates of effect sizes (e.g. Cohen's *d*, Pearson's *r*), indicating how they were calculated

Our web collection on statistics for biologists contains articles on many of the points above.

Software and code

Policy information about availability of computer code

Data collection	Fastp, Bwa mem, Picard, Samtools, RepeatMasker, dfam, Angsd
Data analysis	NgsRelate, genoToVCF, Vcftools, ngsLD, PCAngsd, NGSAdmix, W-IQ-TREE, ModelFinder, dadi, SMC++, GONE, Plink

For manuscripts utilizing custom algorithms or software that are central to the research but not yet described in published literature, software must be made available to editors and reviewers. We strongly encourage code deposition in a community repository (e.g. GitHub). See the Nature Portfolio guidelines for submitting code & software for further information.

Data

Policy information about availability of data

All manuscripts must include a data availability statement. This statement should provide the following information, where applicable:

- Accession codes, unique identifiers, or web links for publicly available datasets
- A description of any restrictions on data availability
- For clinical datasets or third party data, please ensure that the statement adheres to our policy

Raw read data from all individuals are available under NCBI SRA Bioproject PRJNA 1367914. All data to reproduce figures are available at Zenodo (doi: 10.5281/zenodo.18971101). See Supplementary Table 1 for sample information and Supplementary Table 6 for Heterozygosity and inbreeding statistics.

Research involving human participants, their data, or biological material

Policy information about studies with human participants or human data. See also policy information about sex, gender (identity/presentation), and sexual orientation and race, ethnicity and racism.

Reporting on sex and gender	n/a
Reporting on race, ethnicity, or other socially relevant groupings	n/a
Population characteristics	n/a
Recruitment	n/a
Ethics oversight	n/a

Note that full information on the approval of the study protocol must also be provided in the manuscript.

Field-specific reporting

Please select the one below that is the best fit for your research. If you are not sure, read the appropriate sections before making your selection.

☐ Life sciences ☐ Behavioural & social sciences ☒ Ecological, evolutionary & environmental sciences

For a reference copy of the document with all sections, see nature.com/documents/nr-reporting-summary-flat.pdf

Ecological, evolutionary & environmental sciences study design

All studies must disclose on these points even when the disclosure is negative.

Study description	Population genomic analysis of Eastern south pacific Humpback whales on breeding and feeding grounds
Research sample	26 biopsy samples from free-ranging whales
Sampling strategy	Samples were taken from free-ranging whales that showed no visible signs of illness on one breeding ground (Ecuador) and two fedding grounds (Magellan Strait, Antarctic Peninsula).
Data collection	In Antarctic Peninsula, the samples (n = 5) were collected using a Paxarm MK24C remote biopsy system.The biopsies in Ecuador (n = 10) and Magellan Strait (n = 11) were collected using 7 mm diameter dart tips made of surgical steel (Ceta-dart), targeting the upper flank near the dorsal fin propelled by crossbows of 150 and 175 lb, respectively. All skin samples were preserved in 2mL cryotubes containing 95% ethanol and stored at 4°C until DNA extraction.
Timing and spatial scale	Antarctic peninsula: 26/2/2010-5/3/2010; Ecuador: 24/9/2022-1/9/2023; Magellan Strait: 11/2/2022-26/3/2023
Data exclusions	4 specimens were excluded from population genomic inferences, as they comprised first or second degree relatives of other specimens in the sample.
Reproducibility	n/a
Randomization	Data were checked for close relatives. Of any pair of close relatives, one specimen was excluded in order to make the sample a random representation of the respective breeding/feeding ground.
Blinding	Blinding during data acquisition and data processing; blinding for hypothesis-free population genomic inferences.

Did the study involve field work? ☒ Yes ☐ No

Field work, collection and transport

Field conditions	Biopsy sampling from a small boat
Location	Antarctic peninsula, Ecuador, and Magellan Strait (see table S1 for sample-specific coordinates)
Access & import/export	We have complied with all relevant ethical regulations for animal research and followed the Animal Protection Law from Chile and Ecuador, respectively. Humpback whale samples in the Magellan Strait and the Antarctic peninsula were obtained by experienced

researchers following bioethical guidelines of the Comité de Ética, Bioética y Bioseguridad from Universidad de Concepción (protocol number CEBB 1081-2021), Chile. Regarding sampling in the Magellan Strait, the protocol and number of samples were performed according to research permit N°E-2021-531 approved of by the Subsecretaría de Pesca y Acuicultura of Chile. For Ecuador, skin samples were obtained by experienced researchers following the protocol and number of samples according to research permit of the Ministerio del Ambiente, Agua y Transición Ecológica No. 2323 of Ecuador. Moreover, sampling was performed in accordance with the local Forestry and Conservancy of Natural Protected Areas and Wildlife Law, and the guidelines of the general regulation from the Interministerial agreement no. 20140004 from Ecuador.

Disturbance: Disturbance was minimized by targeting the upper flank near the dorsal fin and using an estabilished biopsy technology which minimizes impact.

Reporting for specific materials, systems and methods

We require information from authors about some types of materials, experimental systems and methods used in many studies. Here, indicate whether each material, system or method listed is relevant to your study. If you are not sure if a list item applies to your research, read the appropriate section before selecting a response.

Materials & experimental systems

n/a	Involved in the study	
☒	☐	Antibodies
☒	☐	Eukaryotic cell lines
☒	☐	Palaeontology and archaeology
☐	☒	Animals and other organisms
☒	☐	Clinical data
☒	☐	Dual use research of concern
☒	☐	Plants

Methods

n/a	Involved in the study	
☒	☐	ChIP-seq
☒	☐	Flow cytometry
☒	☐	MRI-based neuroimaging

Animals and other research organisms

Policy information about studies involving animals; ARRIVE guidelines recommended for reporting animal research, and Sex and Gender in Research

Laboratory animals: n/a

Wild animals: Biopsies were taken from free-ranging whales. No animals were captured.

Reporting on sex: Sex was not considered in data analysis, but in interpretation (female philopatry, matrilineal traditions for feeding grounds).

Field-collected samples: Biopsies were taken from free-ranging whales. Details are reported in table S1.

Ethics oversight: We have complied with all relevant ethical regulations for animal research and followed the Animal Protection Law from Chile and Ecuador, respectively. Humpback whale samples in the Magellan Strait and the Antarctic peninsula were obtained by experienced researchers following bioethical guidelines of the Comité de Ética, Bioética y Bioseguridad from Universidad de Concepción (protocol number CEBB 1081-2021), Chile. Regarding sampling in the Magellan Strait, the protocol and number of samples were performed according to research permit N°E-2021-531 approved of by the Subsecretaría de Pesca y Acuicultura of Chile. For Ecuador, skin samples were obtained by experienced researchers following the protocol and number of samples according to research permit of the Ministerio del Ambiente, Agua y Transición Ecológica No. 2323 of Ecuador. Moreover, sampling was performed in accordance with the local Forestry and Conservancy of Natural Protected Areas and Wildlife Law, and the guidelines of the general regulation from the Interministerial agreement no. 20140004 from Ecuador.

Note that full information on the approval of the study protocol must also be provided in the manuscript.

Plants

Seed stocks: n/a

Novel plant genotypes: n/a

Authentication: n/a
